# Identification of GC-rich LAT genes in birds

**DOI:** 10.1371/journal.pone.0283431

**Published:** 2023-04-06

**Authors:** Sarka Janusova, Veronika Krchlikova, Tomas Hron, Daniel Elleder, Ondrej Stepanek

**Affiliations:** 1 Laboratory of Adaptive Immunity, Institute of Molecular Genetics of the Czech Academy of Sciences, Prague, Czech Republic; 2 Faculty of Science, Department of Cell Biology, Charles University, Prague, Czech Republic; 3 Laboratory of Viral and Cellular Genetics, Institute of Molecular Genetics of the Czech Academy of Sciences, Prague, Czech Republic; Chang Gung University, TAIWAN

## Abstract

Linker for activation of T cells (LAT) plays a key role in T-cell antigenic signaling in mammals. Accordingly, LAT orthologues were identified in the majority of vertebrates. However, *LAT* orthologues were not identified in most birds. In this study, we show that *LAT* gene is present in genomes of multiple extant birds. It was not properly assembled previously because of its GC-rich content. LAT expression is enriched in lymphoid organs in chicken. The analysis of the coding sequences revealed a strong conservation of key signaling motifs in LAT between chicken and human. Overall, our data indicate that mammalian and avian *LAT* genes are functional homologues with a common role in T-cell signaling.

## Introduction

Linker for activation of T-cells (LAT) is a key transmembrane adaptor protein in the T-cell antigen receptor (TCR) signaling pathway [1–4]. It consists of a very short extracellular domain, a transmembrane domain with two palmitoylated cysteine residues, and an intracellular tail containing several signaling phosphotyrosine motifs [1, 2, 5, 6].

The importance of LAT was first demonstrated in LAT-deficient Jurkat cell lines, JCaM2 and ANJ3. These cell lines have impaired calcium signaling and ERK phosphorylation after TCR activation [2, 7]. LAT-deficient mice show a severe block in early thymic T-cell development, resulting in a low number of peripheral T cells [4, 8, 9] and defective distal TCR signaling [2, 4, 10].

Four of the nine conserved tyrosine residues (Y132, Y171, Y191, and Y226, numbering according to human LAT throughout the manuscript) in the intracellular part of LAT were identified as important docking sites for several downstream molecules in the TCR signaling cascade, such as Grb2, Gad, and PLCγ1 [1–3, 6, 10–13]. Phosphorylation of these tyrosine residues via ZAP-70 kinase is a crucial step for triggering downstream signaling pathways [1, 2, 13].

Phosphorylated Y132 is the only motif in LAT that recruits PLCγ1. Accordingly, Y132 was shown to be essential for TCR-downstream signaling in Jurkat cells as well as in mice [2, 9, 11–14]. Surprisingly, Y132 is a suboptimal substrate for ZAP-70 due to the glycine residue in position 131 in all known LAT sequences in tetrapods [12, 15]. It has been proposed that inefficient phosphorylation of Y132 by ZAP-70 introduces a delay, which is important for ligand discrimination via a kinetic proofreading mechanism [16]. Some fish species have aspartate or glutamate residue at the position 131, which makes Y132 an optimal substrate for ZAP-70 [12]. The biological significance of the interspecies differences in the amino acid preceding Y132 is unclear, but it has been proposed that some fish may need a negatively charged amino acid at the 131 position to compensate for low kinase activity in a cold environment [12].

LAT deficiency in humans manifests with an early onset of severe combined immunodeficiency (SCID) with recurrent infections [4, 17]. Patients have impaired T-cell function with a skewed response to Th2, reduced number of B cells, and increased number of γδ T cells. These conditions are lethal in the early age of patients unless bone marrow transplantation is performed [17].

LAT was described in tetrapods, fish and in two palaeognath birds (*Dromaius novaehollandiae* and *Apteryx rowi*) [12]. Surprisingly, in the avian clade Neognathae, which contains the vast majority of extant bird species, *LAT* orthologue has not been identified so far [12]. This opened the possibility that T-cell signaling does not rely on this adaptor protein in most birds.

We used our previous experience in the search for avian genes that are difficult to sequence and identify due to their high GC content [18]. In this study, we show that LAT is in fact encoded in the genome of neognath birds including chicken *(Gallus gallus)*. Moreover, avian LAT contains conserved signaling motifs and is prevalently expressed in lymphoid tissues. This strongly suggests that LAT plays the same role in birds as in mammals.

## Material and methods

### Identification and phylogenetic analysis of avian LAT orthologues

For the identification and assembly of avian LAT sequences, we used ‘raw’ next generation sequencing (NGS) data available in the Sequence Read Archive (SRA) of the National Center for Biotechnology information (NCBI). Both RNA-seq and genomic SRA datasets were utilized. Sequencing reads originating from avian LAT were identified by BLASTN searches using various known avian and non-avian LAT sequences as baits. The collected reads of each avian species were consequently assembled using DNASTAR Lasergene SeqMan Pro and CLC genomics workbench software. Nucleotide sequence data reported are available in the Third Party Annotation Section of the DDBJ/ENA/GenBank databases under the accession numbers TPA: BK061375-BK061384 (BK061375 –*Apteryx rowi*, BK061376 –*Struthio camelus*, BK061377 –*Gallus gallus*, BK061378 –*Numida meleagris*, BK061379 –*Meleagris gallopavo*, BK061380 –*Anas platyrhynchos*, BK061381 –*Anser cygnoides*, BK061382 –*Taeniopygia guttata*, BK061383 –*Parus major*, BK061384 –*Pipra filicauda*).

Phylogenetic analysis was conducted for all newly identified avian LAT sequences together with following known vertebrate orthologues: *Homo sapiens* (NCBI RefSeq ID: NP_001014987.1), *Pan troglodytes* (XP_009439105.1), *Mus musculus* (NP_034819.1), *Equus caballus* (XP_023471840.1), *Ornithorhynchus anatinus* (XP_028913760.1), *Anolis carolinensis* (XP_008115721.1), *Alligator mississippiensis* (XP_014453735.1), *Xenopus tropicalis* (XP_031749589.1), *Danio rerio* (NP_001137156.1), *Dromaius novaehollandiae* (XP_025978835.1). Sequence alignment was performed using Mafft v7.487 with default settings. More specifically, amino acid sequences were aligned separately for i) mammals, ii) reptiles, iii) fish and amphibia, and iv) birds. The resulting alignments were merged into a single alignment using MAFFT L-INS-i algorithm. Phylogenetic tree was reconstructed using PhyML with 500 bootstrap replicates, LG substitution model, site rate variation under gamma distribution with 4 categories, and Subtree-Pruning-Regrafting (SPR) searching operations.

### Polymerase chain reaction (PCR)

Total RNA was isolated using TRI reagent (Sigma-Aldrich) from spleen tissue of Brown Leghorn chicken (*Gallus gallus*) within a previous project [19]. The reverse transcription was performed using the SMART RACE (Clontech) procedure. Chicken LAT (cLAT) amplification was performed using a mixture of two polymerases (1:200 Deep Vent: Taq; both from NEB) and primers 5‘-TCCCAAAGGCGGCGGT and 5‘-CGTCTTCTCAGGTTGCGTCAGC. 5 μl of cDNA were used in a 20 μl reaction. The PCR program was set to 95°C for 2 min, followed by 30 cycles of 95°C for 30 s and 65°C for 10 min. The long elongation times allow for efficient amplification of GC-rich sequences. PCR products were separated on 1% agarose gel. Band of interest was purified using QIAEX II Gel Extraction Kit (Qiagen) and submitted for Sanger sequencing (SEQme).

### Relative mRNA expression in different organs

For *in silico* determination of relative expression of cLAT, we employed a series of tissue-specific RNA sequencing datasets, publicly available in SRA under the study ID PRJEB12891. cLAT reads were extracted from individual datasets using BLASTN search with default parameters and bit score threshold 100. Consequently, numbers of extracted reads for each tissue were calculated and expressed as normalized RPKM (Reads per kilobase per million mapped reads) values.

### Ethics statement

There are no relevant ethical concerns in this study. No human subjects were studied and no experiments on animals were performed within this project.

## Results

**Fig 1:**
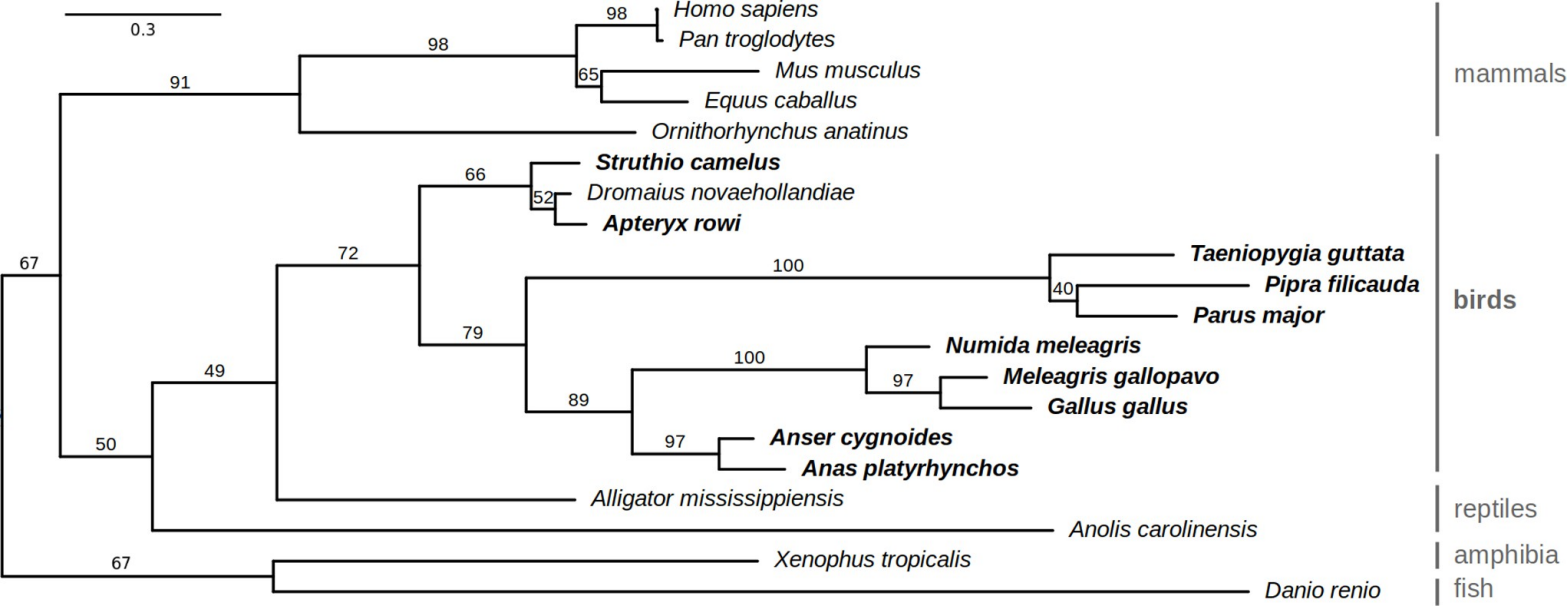
Phylogenetic relationship of *LAT* genes from birds and other vertebrates. The maximum likelihood tree was constructed from the newly identified avian *LAT* sequences together with selected orthologues from other vertebrates. Bootstrap support values (percent of 500 replicates) are shown above the branches. Species with newly identified LAT sequence are in bold. The scale bar above indicates the number of substitutions per site.

To identify *LAT* genes in avian genomes, we used similar approach as in our previous searches for avian GC-rich ‘hidden’ genes [18–20]. We performed homology-based searches of the SRA datasets in NCBI (mostly represented by Illumina RNA-seq data) to assemble full coding sequences of LAT in ten bird species including chicken. To further verify that these represent true *LAT* orthologues, we inferred a phylogenetic relationship with selected vertebrate *LAT* sequences. The clustering of mammalian, avian, and reptilian *LAT* sequences followed known evolutionary relationships (Fig 1).

**Fig 2:**
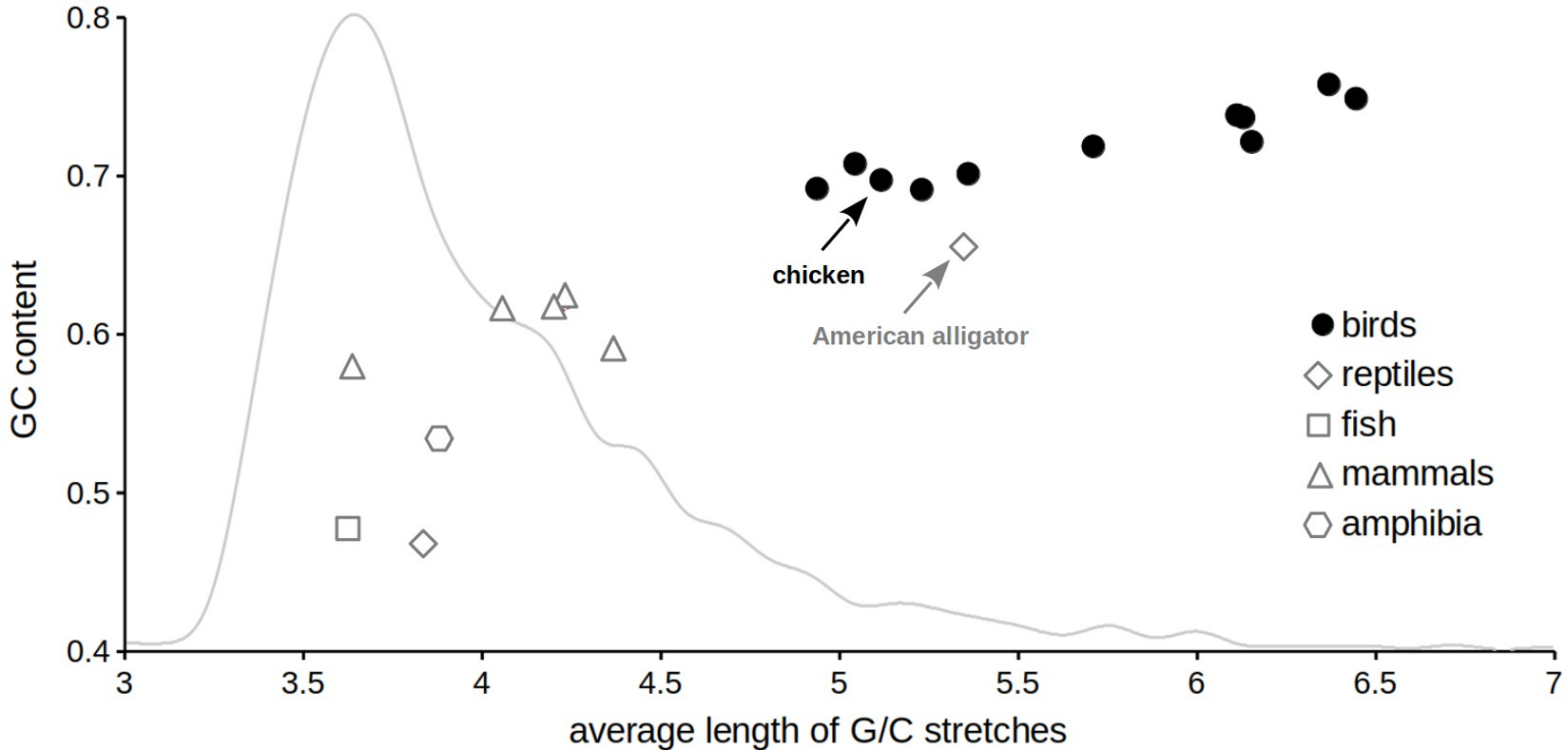
*LAT* gene exhibits a high GC content and long G/C stretches in birds. Comparison of GC content and the presence of G/C stretches in *LAT* genes from birds and other vertebrates. Dot plot was generated based on the coding sequences of *LAT* gene from vertebrate species shown in Fig 1. GC content is plotted against average length of sequence stretches containing G or C nucleotides. G/C-stretch was defined as an undisrupted sequence of at least three consecutive G or C nucleotides [18]. To allow comparison with all annotated chicken genes, a histogram showing the distribution of average G/C-stretch length in the chicken RefSeq gene category is depicted by a gray line. Chicken RefSeq genes comprise the complete set of approximately six thousand chicken coding sequences longer than 299 nucleotides. The chicken (*Gallus gallus*) and American alligator (*Alligator mississippiensis*), which is the closest relative to birds from included reptile species, are highlighted by arrows.

All newly identified avian *LAT* sequences have high GC content and are richer in G/C stretches than their non-avian orthologues (Fig 2). These characteristics are the main cause of their resistance to PCR amplification [18]. To overcome these difficulties, we used our previously optimized PCR conditions (see Methods) to amplify the full-length chicken LAT (cLAT) coding sequence from splenic RNA, which was further confirmed by sequencing.

As a further line of evidence pointing to direct orthology between avian and mammalian *LAT* genes, we could identify synthetic gene order in several species where larger genomic contigs were available. Most clearly annotated example is kiwi (*A*. *rowi*) genomic contig NW_020447579, where *LAT* lies next to SPNS1, a gene whose orthologue is also genomic neighbor of human *LAT*.

**Fig 3:**
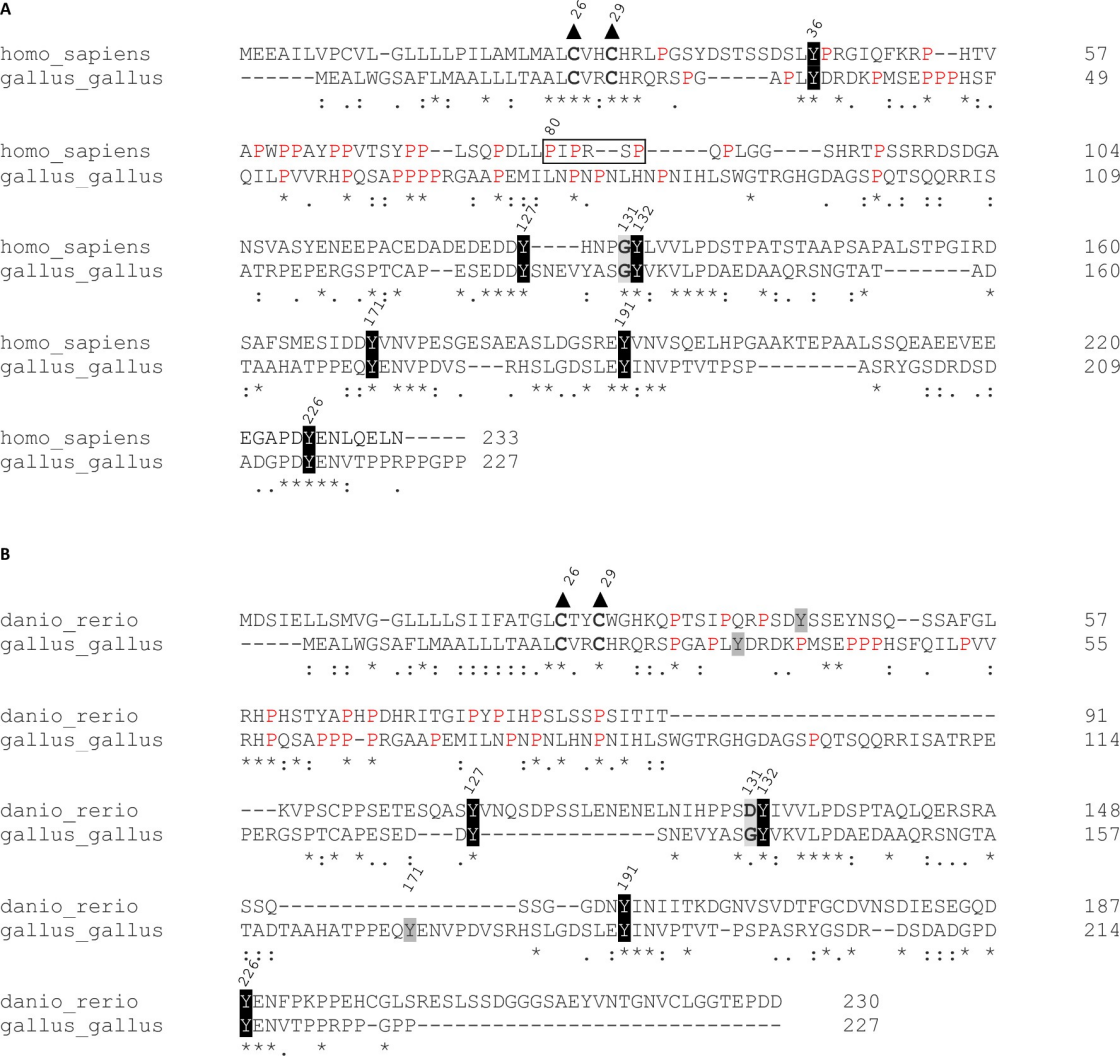
cLAT contains conserved palmitoylation and signaling motifs. The alignments of amino acid LAT sequences of human and chicken (A) and zebrafish and chicken (B) are shown. Conserved tyrosine residues are highlighted in black, glycine and/or aspartate at position 131 is highlighted in grey, conserved palmitoylated cysteines are in bold with black triangle, prolines in the proline-rich sequence (amino acids 30–100 of hLAT) are in red, proline-rich LCK-binding motif is highlighted in the hLAT sequence.

The alignment of the amino acid sequence cLAT with human LAT (hLAT), and zebrafish (*Danio rerio*) LAT (zLAT) revealed that the conserved palmitoylation cysteines, phosphorylation tyrosine motifs [21] are present in all three sequences (Fig 3). cLAT includes the glycine at the position 131, which is present in hLAT, but not in the zLAT, suggesting that the PLCγ-binding tyrosine in cLAT is not an optimal substrate for ZAP70-mediated phosphorylation [12]. cLAT as well as zLAT do not contain a proline-rich motif PIPRSP, which mediates the interaction between LAT and the SH3 domain of LCK and facilitates the down-stream TCR signaling in mammals [21]. Despite this missing motif, all three LAT sequences contain numerous prolines in the N-terminal part of their intracellular domains. Overall, similarities in the conserved amino acid residues suggest that cLAT is a functional orthologue of hLAT and zLAT.

**Fig 4:**
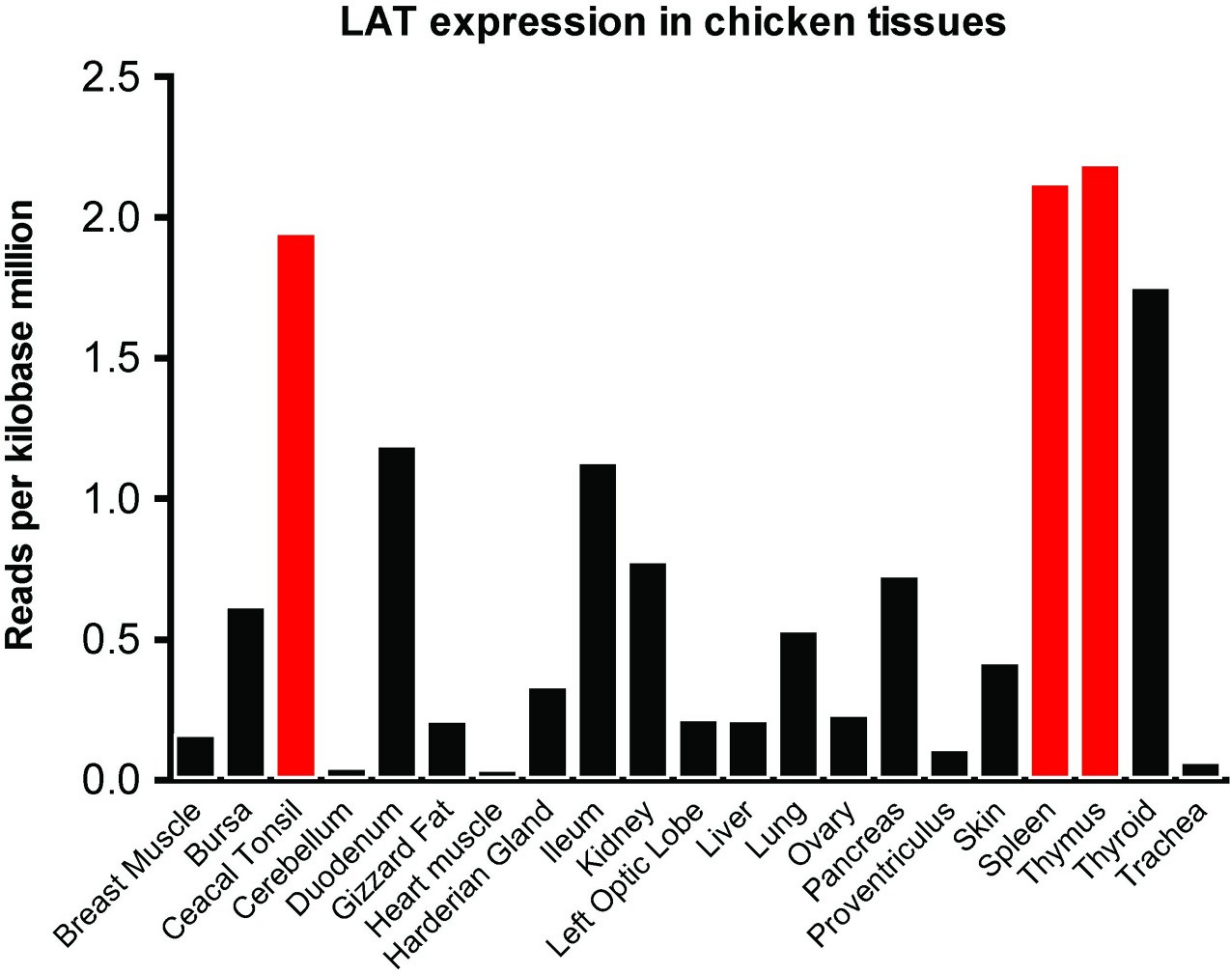
Expression of cLAT is enriched in immune-related organs. The expression levels of cLAT mRNA was determined *in silico* from SRA data (see Methods). Immune-related organs are depicted by red bars.

LAT is mostly expressed in immunological-relevant organs, such as thymus, tonsils, or spleen in mammals [1, 22]. Using the analysis of publicly available transcriptomic data, we documented a similar expression profile in chicken (Fig 4).

## Discussion

LAT plays an instrumental role in the adaptive immune system of mammals. However, the respective gene was not identified in extant birds with the exception of a relatively small clade of Palaeognathae. In this study, we identified *LAT* orthologues in the genome of several birds from the numerous clades of Neognathae, including chicken. This suggests that *LAT* is generally present in avian species. The most probable reason, why *LAT* was not identified in Neognathae birds before, is the high content of GC-rich sequences. These represent the main obstacle in successful amplification and assembly of a subset of avian genes [18, 23–25]. The potential evolutionary cause for this high GC content in avian *LAT* is not clear. Phylogenetic analysis showed that avian *LAT* sequences separate from those present in mammalian, fish or reptilian genomes, as expected from the known evolutionary relationships.

Furthermore, *LAT* expression is enriched in the lymphoid organs in chicken similarly to mammals [26] suggesting a common function in both animal classes. On the protein level, cLAT and hLAT share striking similarities in the functionally relevant parts such as conserved cysteine and tyrosine motifs.

Overall, we conclude that missing LAT in the annotated genomes of neognath birds was caused by technical issues originating from its high GC content. The comparison of the gene expression patterns and the primary structure of the protein products in chicken, human and zebrafish revealed striking similarities. For this reason, we conclude that mammalian, fish and avian *LAT* genes are functional orthologues.

## References

[1] Zhang W., et al., LAT: the ZAP-70 tyrosine kinase substrate that links T cell receptor to cellular activation. Cell, 1998. 92(1): p. 83–92. doi: 10.1016/s0092-8674(00)80901-0 9489702

[2] Wange R.L., LAT, the Linker for Activation of T Cells: A Bridge Between T Cell-Specific and General Signaling Pathways. Science's STKE, 2000. 2000(63): p. re1. doi: 10.1126/stke.2000.63.re1 11752630

[3] Stepanek O., Draber P., and Horejsi V., Palmitoylated transmembrane adaptor proteins in leukocyte signaling. Cell Signal, 2014. 26(5): p. 895–902. doi: 10.1016/j.cellsig.2014.01.007 24440308

[4] Bacchelli C., et al., Mutations in linker for activation of T cells (LAT) lead to a novel form of severe combined immunodeficiency. J Allergy Clin Immunol, 2017. 139(2): p. 634–642 e5. 10.1016/j.jaci.2016.05.036 27522155

[5] Zhang W., Trible R.P., and Samelson L.E., LAT palmitoylation: its essential role in membrane microdomain targeting and tyrosine phosphorylation during T cell activation. Immunity, 1998. 9(2): p. 239–46. doi: 10.1016/s1074-7613(00)80606-8 9729044

[6] Weber J.R., et al., Molecular cloning of the cDNA encoding pp36, a tyrosine-phosphorylated adaptor protein selectively expressed by T cells and natural killer cells. The Journal of experimental medicine, 1998. 187(7): p. 1157–1161. doi: 10.1084/jem.187.7.1157 9529333 PMC2212210

[7] Finco T.S., et al., LAT Is Required for TCR-Mediated Activation of PLCγ1 and the Ras Pathway. Immunity, 1998. 9(5): p. 617–626. 9846483 10.1016/s1074-7613(00)80659-7

[8] Zhang W., et al., Essential Role of LAT in T Cell Development. Immunity, 1999. 10(3): p. 323–332. doi: 10.1016/s1074-7613(00)80032-1 10204488

[9] Aguado E., et al., Induction of T Helper Type 2 Immunity by a Point Mutation in the LAT Adaptor. Science (New York, N.Y.), 2002. 296: p. 2036–40. doi: 10.1126/science.1069057 12065839

[10] Lin J. and Weiss A., Identification of the Minimal Tyrosine Residues Required for Linker for Activation of T Cell Function. Journal of Biological Chemistry, 2001. 276(31): p. 29588–29595. doi: 10.1074/jbc.M102221200 11395491

[11] Malbec O., et al., Linker for activation of T cells integrates positive and negative signaling in mast cells. J Immunol, 2004. 173(8): p. 5086–94. doi: 10.4049/jimmunol.173.8.5086 15470052

[12] Lo W.L., et al., Slow phosphorylation of a tyrosine residue in LAT optimizes T cell ligand discrimination. Nat Immunol, 2019. 20(11): p. 1481–1493. doi: 10.1038/s41590-019-0502-2 31611699 PMC6858552

[13] Paz P.E., et al., Mapping the Zap-70 phosphorylation sites on LAT (linker for activation of T cells) required for recruitment and activation of signalling proteins in T cells. The Biochemical journal, 2001. 356(Pt 2): p. 461–471. doi: 10.1042/0264-6021:3560461 11368773 PMC1221857

[14] Malissen B., et al., Integrative biology of T cell activation. Nat Immunol, 2014. 15(9): p. 790–7. doi: 10.1038/ni.2959 25137453

[15] Shah N.H., et al., An electrostatic selection mechanism controls sequential kinase signaling downstream of the T cell receptor. eLife, 2016. 5: p. e20105. doi: 10.7554/eLife.20105 27700984 PMC5089863

[16] McKeithan T.W., Kinetic proofreading in T-cell receptor signal transduction. Proceedings of the National Academy of Sciences, 1995. 92(11): p. 5042–5046. doi: 10.1073/pnas.92.11.5042 7761445 PMC41844

[17] Keller B., et al., Early onset combined immunodeficiency and autoimmunity in patients with loss-of-function mutation in LAT. J Exp Med, 2016. 213(7): p. 1185–99. doi: 10.1084/jem.20151110 27242165 PMC4925012

[18] Hron T., et al., Hidden genes in birds. Genome biology, 2015. 16(1): p. 164–164. doi: 10.1186/s13059-015-0724-z 26283656 PMC4539667

[19] Rohde F., et al., Characterization of Chicken Tumor Necrosis Factor-α, a Long Missed Cytokine in Birds. Front Immunol, 2018. 9: p. 605. 29719531 10.3389/fimmu.2018.00605 PMC5913325

[20] Burkhardt N.B., et al., The Discovery of Chicken Foxp3 Demands Redefinition of Avian Regulatory T Cells. J Immunol, 2022. 208(5): p. 1128–1138. doi: 10.4049/jimmunol.2000301 35173035

[21] Lo W.-L., et al., Lck promotes Zap70-dependent LAT phosphorylation by bridging Zap70 to LAT. Nature Immunology, 2018. 19(7): p. 733–741. doi: 10.1038/s41590-018-0131-1 29915297 PMC6202249

[22] Fagerberg L., et al., Analysis of the human tissue-specific expression by genome-wide integration of transcriptomics and antibody-based proteomics. Mol Cell Proteomics, 2014. 13(2): p. 397–406. doi: 10.1074/mcp.M113.035600 24309898 PMC3916642

[23] Huttener R., et al., Sequencing refractory regions in bird genomes are hotspots for accelerated protein evolution. BMC Ecol Evol, 2021. 21(1): p. 176. doi: 10.1186/s12862-021-01905-7 34537008 PMC8449477

[24] Botero-Castro F., et al., Avian Genomes Revisited: Hidden Genes Uncovered and the Rates versus Traits Paradox in Birds. Mol Biol Evol, 2017. 34(12): p. 3123–3131. doi: 10.1093/molbev/msx236 28962031

[25] Beauclair L., et al., Sequence properties of certain GC rich avian genes, their origins and absence from genome assemblies: case studies. BMC Genomics, 2019. 20(1): p. 734. doi: 10.1186/s12864-019-6131-1 31610792 PMC6792250

[26] Facchetti F., et al., Linker for Activation of T Cells (LAT), a Novel Immunohistochemical Marker for T Cells, NK Cells, Mast Cells, and Megakaryocytes: Evaluation in Normal and Pathological Conditions. The American Journal of Pathology, 1999. 154(4): p. 1037–1046. doi: 10.1016/S0002-9440(10)65356-4 10233842 PMC1866564

