# Supplementary material for: Identification of GC-rich LAT genes in birds
Source: PLoS One. 2023 Apr 6;18(4):e0283431. doi: 10.1371/journal.pone.0283431 (PMC10079008; doi:10.1371/journal.pone.0283431)
Supplement: Attachment — Submitted filename: point by point response.pdf [file pone.0283431.s001.pdf]

Point by point response to the Reviewers

Journal Requirements:

When submitting your revision, we need you to address these additional requirements.

1. Please ensure that your manuscript meets PLOS ONE's style requirements, including those for file naming. The PLOS ONE style templates can be found at

https://journals.plos.org/plosone/s/file?id=wjVg/PLOSOne_formatting_sample_main_body.pdf and

https://journals.plos.org/plosone/s/file?id=ba62/PLOSOne_formatting_sample_title_authors_affiliations.pdf.

We formatted the manuscript accordingly.

2. Thank you for stating the following in the Acknowledgments Section of your manuscript:

"This project was supported by the National Institute of virology and bacteriology (Programme EXCELES, ID Project No. LX22NPO5103) - Funded by the European Union - Next Generation EU and Charles University Grant Agency (Project No. 984120 to SJ)."

We note that you have provided funding information that is not currently declared in your Funding Statement. However, funding information should not appear in the Acknowledgments section or other areas of your manuscript. We will only publish funding information present in the Funding Statement section of the online submission form.

Please remove any funding-related text from the manuscript and let us know how you would like to update your Funding Statement. Currently, your Funding Statement reads as follows:

"This project was supported by the National Institute of virology and bacteriology (Programme EXCELES, ID Project No. LX22NPO5103) - Funded by the European Union - Next Generation EU (to OS) and Charles University Grant Agency (Project No. 984120 to SJ)."

Please include your amended statements within your cover letter; we will change the online submission form on your behalf.

Please, include the following Financial Statement ""This project was supported by the National Institute of virology and bacteriology (Programme EXCELES, ID Project No. LX22NPO5103) - Funded by the European Union - Next Generation EU and Charles University Grant Agency (Project No. 984120 to SJ)".

We deleted this from the Acknowledgement section and included it in the Cover letter.

3. Please include your full ethics statement in the 'Methods' section of your manuscript file. In your statement, please include the full name of the IRB or ethics committee who approved or waived your study, as well as whether or not you obtained informed written or verbal consent. If consent was waived for your study, please include this information in your statement as well.

We added the full Ethics Statement in the manuscript, although there are no relevant ethical concerns.

4. Please review your reference list to ensure that it is complete and correct. If you have cited papers that have been retracted, please include the rationale for doing so in the manuscript text, or remove these references and replace them with relevant current references. Any changes to the reference list should be mentioned in the rebuttal letter that accompanies your revised manuscript. If you need to cite a retracted article, indicate the article's retracted status in the References list and also include a citation and full reference for the retraction notice.

We reviewed the reference list.

Reviewer 1

LAT is a critical signaling adapter molecule in the T cell receptor signaling pathway that very likely plays a role in ligand discrimination. LAT orthologues have been identified in various species, including fish. Reports on LAT in bird species have been lacking.

In the submitted manuscript, the authors capitalize on their experience searching for and analyzing genes with high GC content. The authors describe the identification of chicken LAT and postulate – based on sequence homology to human LAT and chicken LAT expression – that chicken LAT full fills a similar function as mammalian in LAT in T cell signaling.

In sum, this is a clearly presented, brief definitive report on the identification of chicken LAT that is informative for the T cell signaling community.

We are thankful for the positive evaluation of the manuscript and useful comments.

The authors should enhance the manuscript in a couple of manners:

1. The presentation of the amino acid sequence alignment in figure 3 can be improved. It should contain the numbering of e.g. tyrosine residues that are mentioned in the text.

We added the numbering of the tyrosines.

2. The authors should also perform a comparison between chicken and fish LAT (with potential focus on the region around Y132)

We added the comparison of LAT from chicken and zebrafish (Fig. 3B).

3. LAT also contains a proline rich region (the senior author co-published on this). The proline rich region should also be covered in their comparison of different LAT orthologues.

We highlighted the proline-rich motif mediating LCK binding in the human sequence and also highlighted all prolines in the proline-rich sequence. It is interesting that the LCK-binding motif (PIPRSP) is missing in the chicken sequence, but there are still many prolines in the N-terminal part of the LAT intracellular sequence. We comment on this in the revised version of the manuscript.
